# Monitoring prostate cancer under androgen-deprivation therapy: insights from the implementation of a clinical decision support system dashboard

**DOI:** 10.3389/fonc.2026.1746456

**Published:** 2026-03-30

**Authors:** Álvaro Martínez-Pérez, Gonzalo Collantes, José‑Luis Ruiz‑Cerdá, Antonio Conde-Moreno, Regina Gironés, Beatriz Garcillán, Julià Amengual, María Eugenia Gas

**Affiliations:** 1 Plataforma de Big Data, IA, Bioestadística y Bioinformática, Instituto de Investigación Sanitaria La Fe (IIS La Fe), Valencia, Spain; 2 Unidad Mixta de Investigacion en TICs aplicadas a la Reingeniería en Procesos Sociosanitarios, Instituto de Investigación Sanitaria La Fe (IIS La Fe), Valencia, Spain; 3 Servicio de Urología, Hospital Universitario y Politécnico La Fe, Valencia, Spain; 4 Servicio de Oncología Radioterápica, Hospital Universitario y Politécnico La Fe, Valencia, Spain; 5 Servicio de Oncología Médica, Hospital Universitario y Politécnico La Fe, Valencia, Spain; 6 The Medical Affairs Department, Johnson & Johnson, Madrid, Spain

**Keywords:** castration−resistant prostate cancer, clinical decision support system (CDSS), dashboard, prostate cancer, PSA, valued-based medicine

## Abstract

**Introduction:**

Clinical Decision Support Systems (CDSS) assist clinicians in making informed decisions based on clinical guidelines and comprehensive data. In prostate cancer (PC), particularly in patients receiving androgen deprivation therapy (ADT), early detection of castration-resistant prostate cancer (CRPC) and close monitoring of progression biomarkers are essential. This study evaluated the impact of a CDSS on the monitoring and clinical management of advanced PC patients in a hospital setting.

**Methods:**

A retrospective, single-centre cohort study was conducted using structured patient-level data from the institutional Data Warehouse of Hospital La Fe (Valencia, Spain). The study included patients diagnosed with PC who received ADT and were analysed over two consecutive one-year periods: 15 September 2021 to 14 September 2022 (pre-implementation) and 15 September 2022 to 15 September 2023 (post-implementation of the CDSS). Primary outcomes included CRPC detection rates, classification of PSA kinetics instability, and use of palliative radiotherapy.

**Results:**

CDSS implementation was associated with higher CRPC detection rates (from 20.7% to 27.9%). The proportion of patients with unstable PSA kinetics increased after implementation (81.1% vs. 64.7%; p = 0.01). Although the increase in the use of palliative radiotherapy did not reach statistical significance, a modest rise was observed (from 11.7% to 15.1%).

**Discussion:**

The introduction of the CDSS was associated with improved monitoring signals and earlier recognition of disease progression in ADT-treated patients. The system supported enhanced PSA-based risk stratification and more timely multidisciplinary coordination. Although longer follow-up and broader implementation are needed to confirm trends, the findings highlight the potential of digital tools to optimize routine PC management.

## 1 Introduction

Prostate cancer (PC) is the most common male malignancy in Europe and constitutes an important clinical and organizational challenge for public healthcare (1). In Spain, it accounts for about 13% of newly diagnosed male cancers, with over 35,000 new diagnoses annually (2). The overall survival of PC is generally high when the disease is well localized. However, its management across the entire clinical course—from diagnosis to potential progression—requires continuous multidisciplinary coordination across specialties such as Urology, Medical Oncology and Radiation Oncology, among others, to ensure high-quality clinical decision-making.

While localized PC is often associated with favorable outcomes, advanced stages of the disease require systemic therapeutic strategies. It is well known that tumor growth is largely driven by androgen signalling (3). Therefore, androgen deprivation therapy (ADT), in combination with other treatments depending on the disease stage, remains the backbone of treatment for advanced or metastatic PC, aimed to suppress disease activity (4). Although initially effective, a subset of patients under ADT eventually develop resistance, leading to the emergence of castration-resistant prostate cancer (CRPC) (5). This stage of the disease is defined by a continuous rise in prostate-specific antigen (PSA) levels despite achieving castrate levels of testosterone, indicating a transition to a more advanced disease state characterized by a worse prognosis that demands more complex therapeutic approaches (6, 7).

Against this background, the contemporary standard of care for metastatic hormone−sensitive prostate cancer has shifted toward early treatment intensification. Current guidelines recommend combination systemic therapy with ADT plus an androgen−receptor pathway inhibitor (ARPI), with consideration of triplet therapy including docetaxel in appropriate candidates (8). Pivotal randomized trials, ARASENS (ADT, docetaxel, darolutamide) and PEACE−1 (ADT, docetaxel, abiraterone), demonstrated significant overall−survival benefits and delayed progression, supporting triplet therapy in selected patients and underscoring that intensified regimens can alter PSA kinetics and extend the time to CRPC (9, 10).

In routine clinical practice, patients treated with ADT require close monitoring of PSA levels throughout follow-up, as PSA dynamics are critical for detecting disease progression early and intervening promptly when abnormalities arise or when PSA monitoring does not adhere to clinical guidelines. However, current clinical practice often suffers from a lack of uniformity in how patients are managed and disparities in the information available across services, which may delay appropriate clinical decisions (5). Moreover, adherence to clinical practice guidelines is frequently suboptimal, further contributing to variability in care and missed opportunities for timely intervention (11). To help close these gaps at our institution, an interactive hospital-wide dashboard for PC follow-up (MAPA project) was created to provide professionals with a shared, up-to-date view of each patient and of the overall cohort across Urology, Medical Oncology and Radiation Oncology. Building on this foundation, the ARCHIMEDES project extended MAPA into a clinical decision support system that couples routinely collected data with guideline−aligned logic to facilitate consistent, evidence-based decisions (12, 13).

We aimed to explore whether the implementation of the CDSS is associated with changes in the detection and management of disease progression among patients under ADT. The analysis focuses on evaluating potential variations in the identification of CRPC, the frequency of abnormal PSA fluctuation alerts, and the timeliness of clinical interventions. These findings may provide insights into the role of CDSS tools in supporting clinical decision-making, promoting adherence to practice guidelines, and enhancing patient-centered care, while also highlighting their potential to reduce variability in clinical practice, improve coordination among multidisciplinary teams, and foster a data-driven culture that optimizes resource utilization and overall healthcare efficiency.

## 2 Methods

With the aim to evaluate the impact of implementing a CDSS on the follow-up and clinical characterization of PC patients undergoing ADT, the analysis focused on comparing monitoring practices and the progression to CRPC during the period before and after CDSS implementation. To achieve this, we conducted a non-interventional, retrospective, single-center study based on pseudonymized data extracted from the Electronic Health Records (EHRs) of the Valencia La Fe Health Department. The study cohort consisted of patients diagnosed with PC who were being monitored in a hospital setting. Inclusion in the study did not affect patients’ routine medical care or their rights to access healthcare services. The decision to clinically follow−up these patients had been made independently of their inclusion in this analysis. This study was conducted in accordance with the principles outlined in the Declaration of Helsinki and was approved by the relevant institutional ethics committee.

### 2.1 Inclusion criteria

The final study population consisted of all patients diagnosed with PC and assigned to the Valencia La Fe Health Department up to September 15, 2023, who were under follow-up by the Radiation Oncology, Urology, or Medical Oncology services. Eligible patients had to be receiving hormonal treatment with or without antiandrogens (see **Supplementary Table 1**) between September 15, 2021, and September 15, 2023. In addition, they were required to meet at least one of the following conditions: histopathological confirmation of PC diagnosis; or the presence of a diagnostic ICD code for PC (ICD-9: 185; ICD-10: C61) along with at least one of the following procedures—radical prostatectomy (ICD-9: 60.5; ICD-10: 0VT00ZZ, 0VT04ZZ, 0VT07ZZ, 0VT08ZZ), radiotherapy (see **Supplementary Table 2**), or chemotherapy (see **Supplementary Table 3**). Patients were also required to have PSA level monitoring, defined as at least one PSA measurement per year, and not to have participated in a clinical trial.

### 2.2 Timeframe

**Figure 1:**
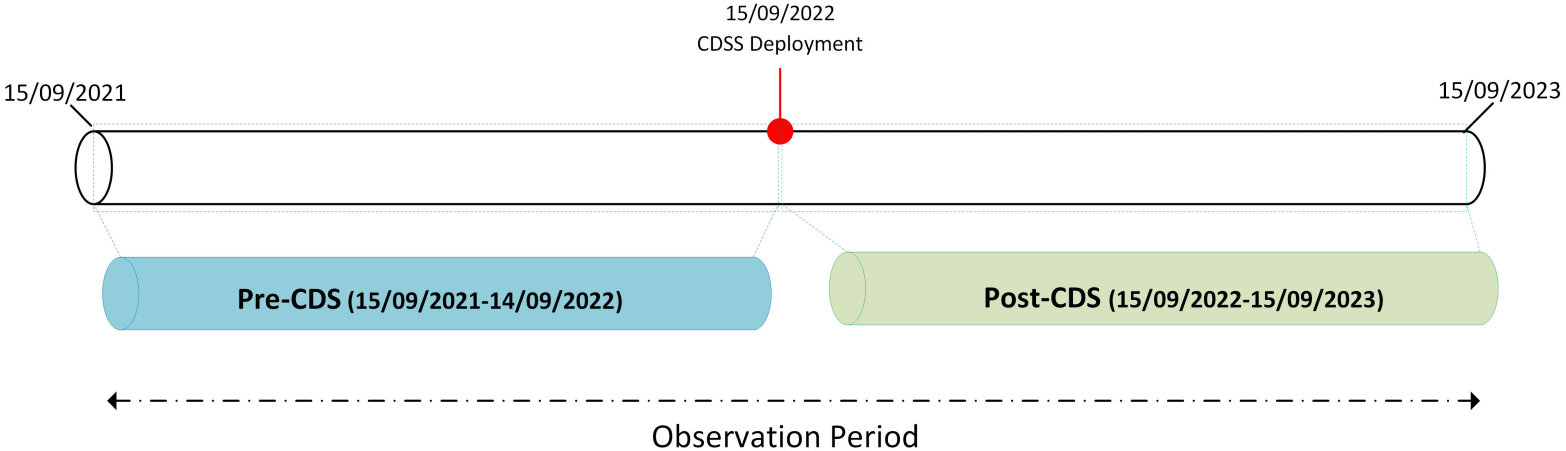
Study timeline illustrating the CDSS deployment and analysis periods. The study was divided into two 12-month intervals: Pre-CDSS (September 15, 2021, to September 14, 2022) and Post-CDSS (September 15, 2022, to September 15, 2023), with CDSS implementation at the midpoint. The two-year observation period enabled comparison of clinical indicators before and after system deployment.

Data were collected over a 24-month observation period, spanning from September 15, 2021, to September 15, 2023. This timeframe was divided into two distinct 12-month intervals: the Pre-CDSS period (September 15, 2021 – September 14, 2022) and the Post-CDSS period (September 15, 2022 – September 15, 2023). The CDSS was deployed on September 15, 2022 (**Figure 1**).

### 2.3 Data source

Data were extracted from the Data Warehouse (DW) of the Big Data, AI, Biostatistics and Bioinformatics Platform at IIS La Fe. This DW contains clinical, epidemiological, and biological information at the individual level for the population cohort assigned to Valencia La Fe Health Department. The dataset includes both structured and unstructured clinical records, such as hospital discharge summaries, pharmacy reports, outpatient consultations, laboratory tests, pathology and radiology reports. A total of 75,000 PC-related diagnoses and procedures coded according to the International Classification of Diseases (ICD-9 and ICD-10) were analyzed.

Artificial intelligence and natural language processing were not used. All study variables and endpoints were obtained from structured EHR data. Unstructured notes were not parsed; when verification of first−line treatment was required, deterministic keyword queries were executed within the EHR using exact string matching.

### 2.4 Dashboard-based monitoring framework

To support the evaluation and follow-up of patients diagnosed with PC, a CDSS dashboard was developed and implemented as part of the MAPA and ARCHIMEDES projects. This tool integrates a comprehensive set of indicators and variables drawn from the DW of the Big Data, AI, Biostatistics, and Bioinformatics Platform at IIS La Fe. It supports real-time monitoring and retrospective analysis of clinical care, disease progression, and healthcare resource utilization for PC patients in the La Fe Health department, and it is fully integrated into the clinical environment routinely used by healthcare professionals. The system performs automatic daily data updates, ensuring that information is accurate and up to date. This direct integration into the physicians’ interface facilitates immediate access to relevant clinical data and supports evidence-based decision-making, ultimately contributing to improved patient care and outcomes. The development and implementation of the CDSS were carried out in close collaboration with the hospital’s Department of Information Technology, ensuring technical reliability and seamless integration within existing workflows.

#### 2.4.1 Access and collaborative use

The dashboard provides centralized, accessible information to all services involved in the follow-up of PC patients (Urology, Medical Oncology and Radiation Oncology). This cross-functional visibility promotes shared decision-making, enhances coordination among care teams and supports continuous improvement in clinical practice through real-time insights.

#### 2.4.2 Structure and domains

The dashboard is structured around three core domains:

- Epidemiological and clinical characterization: Tracks hospital-attended PC patients, focusing on prevalence and incidence of key clinical phenotypes. Baseline variables such as age, PSA, Gleason score, and initial treatment are recorded to support clinical stratification and disease profiling.
- Healthcare resource utilization: Captures the average number of visits to Urology, Medical Oncology, Radiation Oncology, and Primary Care consultations; the proportion of patients requiring hospitalizations or emergency care; and additional metrics such as the average number of imaging studies and laboratory tests performed per patient over the past year, reflecting the intensity of clinical monitoring.
- Quality of care and clinical practice indicators: Assesses adherence to recommended practices and the impact of the dashboard on care quality, including the proportion of CRPC patients without imaging at diagnosis, lack of imaging at ADT initiation, absence of biopsy in newly diagnosed patients, and presence of bone density testing in patients under ADT. Monitoring also covered follow-up practices post-treatment (e.g., PSA testing after radical prostatectomy or radiotherapy), and early detection of biochemical recurrence.

Operational elements are visible to clinicians. At the point of care, the application foregrounds the elements that drive day to day follow−up decisions under ADT. For each patient, it displays the date of the most recent PSA and testosterone tests, the time elapsed since those measurements, longitudinal PSA trends, and a concise clinical profile; at the cohort level, it summarizes characteristics such as age distribution and comorbidities and records the timing and type of treatments administered. The system also flags delays and missing information through clear alerts, which supports prompt remediation of gaps. These features enhance the transparency and efficiency of patient monitoring and promote evidence−based decision making across services.

### 2.5 Clinical alerts: definitions and algorithm implementation

As part of the CDSS embedded in the PC dashboard, a series of automated alerts were developed to enhance patient monitoring, promote adherence to clinical guidelines, and support early clinical intervention. The alert engine was implemented as a rule−based, guideline−aligned decision tree; no machine−learning methods were applied. Alerts are triggered by predefined clinical criteria and temporal thresholds. A key component of this system is an algorithm that monitors the adherence to follow-up protocols, such as timing of testosterone and PSA measurements, and flags deviations from recommended care timelines. These deviations trigger alerts in the monitoring system are shown in **Table 1**.

**Table 1:** Definitions and activation logic of clinical alerts for prostate cancer patients receiving ADT.

Alert	Name	Definition	T_0_ (start time)	T_1_ (end time)
1	No Testosterone measurement available	No testosterone test performed within 90 days of ADT initiation.	Day 90 after ADT start	Date of first testosterone test
2	Testosterone measurement ≥ 50 ng/dL	Testosterone level ≥ 50 ng/dL after 90 days of ADT initiation.	Date of testosterone ≥ 50 ng/dL	Date of testosterone < 50 ng/dL
3	No PSA Measurement available	No PSA test performed within 90 days of ADT initiation.	Day 90 after ADT start	Date of first PSA test
4	PSA not performed within the recommended timeframe	PSA not performed within recommended follow-up (3 or 6 months depending on previous PSA).	Expected PSA follow-up date	Actual PSA test date
5	Patients at risk of CRPC	Patient on ADT showing PSA progression while testosterone remains castrate.	Date when risk criteria are met	Date of confirmed CRPC
6	Stable Patients	Post-treatment PSA stable (SD < 2 ng/mL in last 3 tests).	Start of 3-test stable window	Remains active as long as stable
7	Unstable Patients	Post-treatment PSA irregular (SD ≥ 2 ng/mL in last 3 tests).	Start of 3-test irregular window	Remains active as long as irregular
8	Patients diagnosed with CRPC	Patient meets definition of CRPC (PSA rise + castrate T).	Date of CRPC classification	Date of alert computation

**Figure 2:**
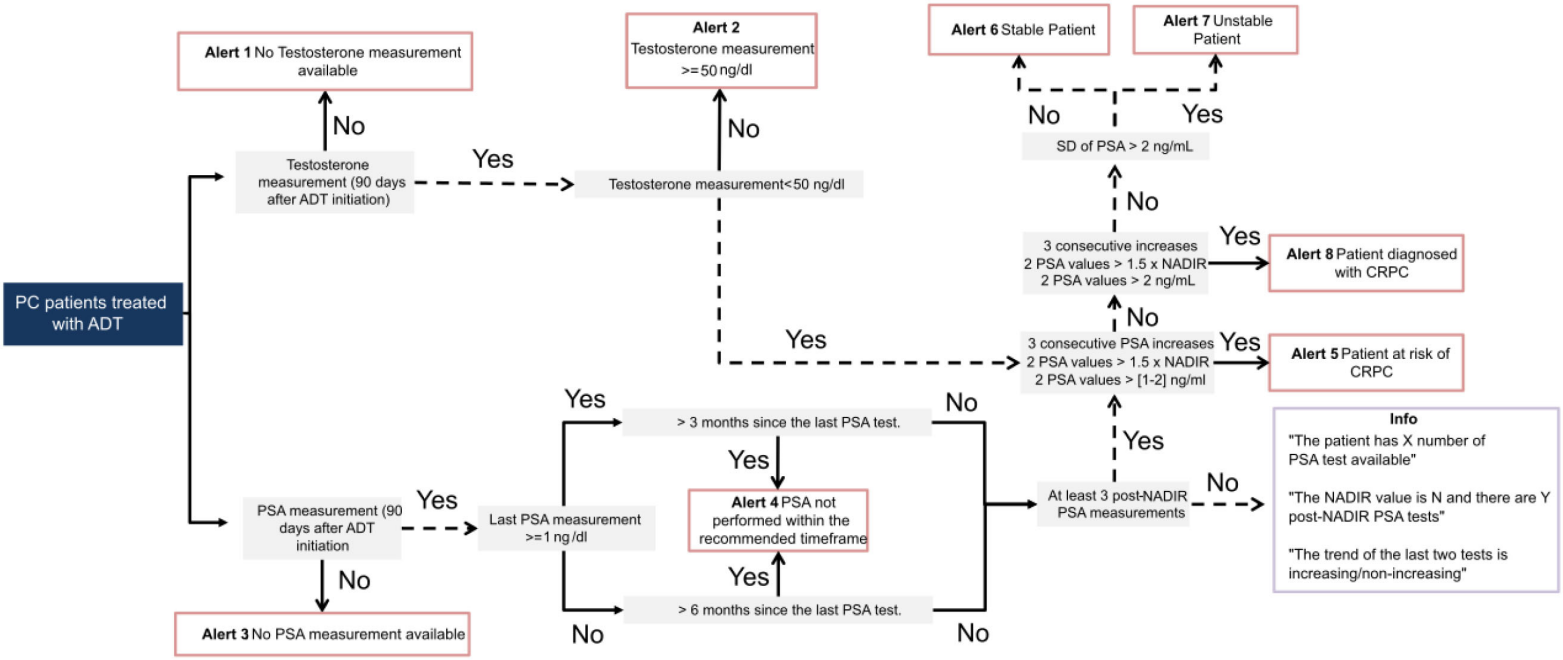
Algorithm for clinical alert generation in prostate cancer patients undergoing ADT. This flowchart illustrates the logic implemented in the CDSS dashboard to monitor patients with PC on ADT. The algorithm evaluates the availability and timing of testosterone and PSA measurements and triggers alerts based on deviations from guideline-recommended follow-up intervals and patterns indicative of disease progression. Alerts 1–4 identify patients with missing or delayed biomarker assessments. Alerts 5 and 8 flag patients who are at risk of, or meet criteria for, CRPC based on PSA kinetics under castrate testosterone levels. Alerts 6 and 7 assess PSA stability over time using the standard deviation of the three most recent post-nadir PSA values. The dashboard also displays supporting information, including the number of PSA tests, nadir value, and PSA trends to aid clinical interpretation.

**Figure 3:**
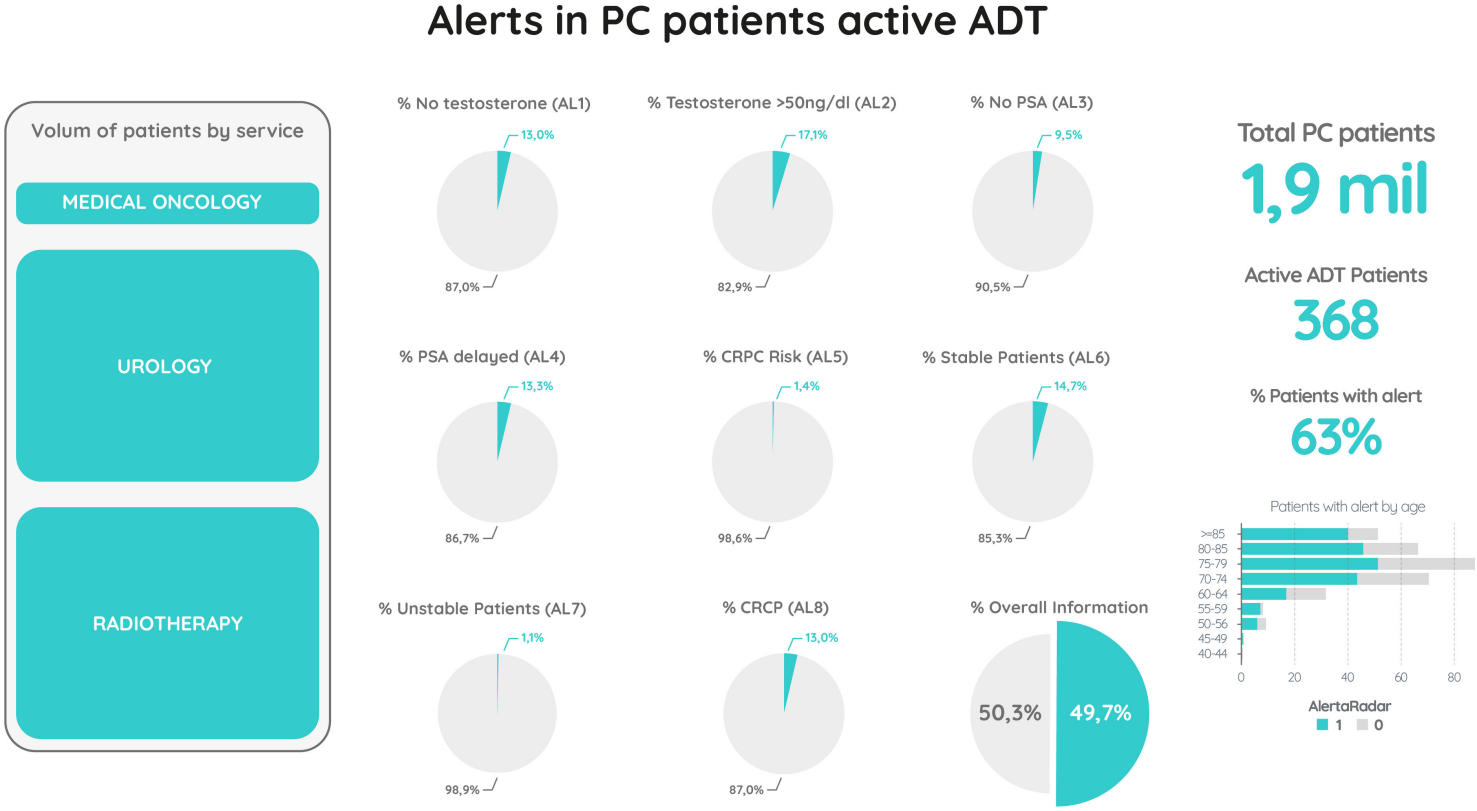
Clinical decision support dashboard for prostate cancer patients under active ADT. This dashboard provides a real-time overview of patients under ADT, highlighting alerts related to treatment monitoring and risk of progression. Image shown for illustrative purposes only.

Alert logic and scope. Alert definitions and activation logic are specified in **Table 1** and implemented by the rule−based decision tree shown in **Figure 2**, which together serve as the authoritative specification. The operational dashboard implementing this decision tree (**Figure 2**) is depicted in Figure 3, providing a cohort‑level overview and alert distribution used in routine monitoring. In brief, the engine verifies timely availability of testosterone and PSA after ADT initiation and flags deviations; if a testosterone result is ≥ 50 ng/dL (EAU−aligned castration threshold (14)), Alert 2 is issued and progression assessment is deferred until castrate levels are confirmed; monitoring intervals for PSA are checked against guideline thresholds; and, in patients with confirmed castrate testosterone and sufficient post−nadir PSA data, stability, instability, risk of progression and castration−resistant status are classified as per **Table 1**. PSA instability (Alert 7)—defined as SD ≥ 2 ng/mL across the last three post−nadir PSA tests—functions as an operational screening flag to prompt guideline−concordant monitoring; it is interpreted with treatment status and testosterone and does not define risk or progression (which remain specified per **Table 1**, **Figure 2**, PCWG3−aligned). CRPC and all alert endpoints were computed solely from structured PSA kinetics and testosterone results; imaging findings did not trigger alerts nor contribute to CRPC classification.

This algorithm provides a robust, guideline-informed framework for identifying patients requiring closer monitoring or therapeutic adjustment. It is implemented as a rule-based decision tree and aligns with international guideline definitions, including those from the European Association of Urology (EAU) (13).

General training sessions on the use of the algorithm integrated within the CDSS were conducted for each participating department (Urology, Radiation Oncology, and Medical Oncology). Follow-up sessions and specialized training activities were also provided for key users, corresponding to the heads of each participating department, who were actively involved in the design, validation of clinical alerts and instruction of the clinicians within their departments on both operational and clinical value of the tool. This fostered greater acceptance and adherence to the system by engaging end users from the early stages of the project. As the platform was highly visual and intuitive, no extensive training was required, further facilitating its adoption in routine clinical practice.

### 2.6 Statistical analysis

Data analysis was conducted using SAS Enterprise Guide and R. Chi-squared tests were employed to compare categorical variables (e.g., proportions). A p-value < 0.05 was considered statistically significant for all analyses.

Continuous variables were compared between periods using the Wilcoxon rank−sum test, and Fisher’s exact test was used when expected frequencies in categorical tables were small. Standardized mean differences were reported for baseline variables as a complementary measure of balance. As a parsimonious multivariable appraisal of baseline comparability, a logistic regression model was fitted with the study period as the outcome, contrasting the Post−CDSS against the Pre−CDSS cohort, and with baseline PSA and time from diagnosis to ADT as continuous predictors and biopsy Gleason as an ordinal predictor. Models were estimated by maximum likelihood on complete−case data and were evaluated using likelihood−ratio tests and McFadden’s pseudo−R². No imputation was performed. The distribution of initial systemic regimens at ADT start was compared analogously using chi−squared or Fisher’s exact tests.

## 3 Results

**Figure 4:**
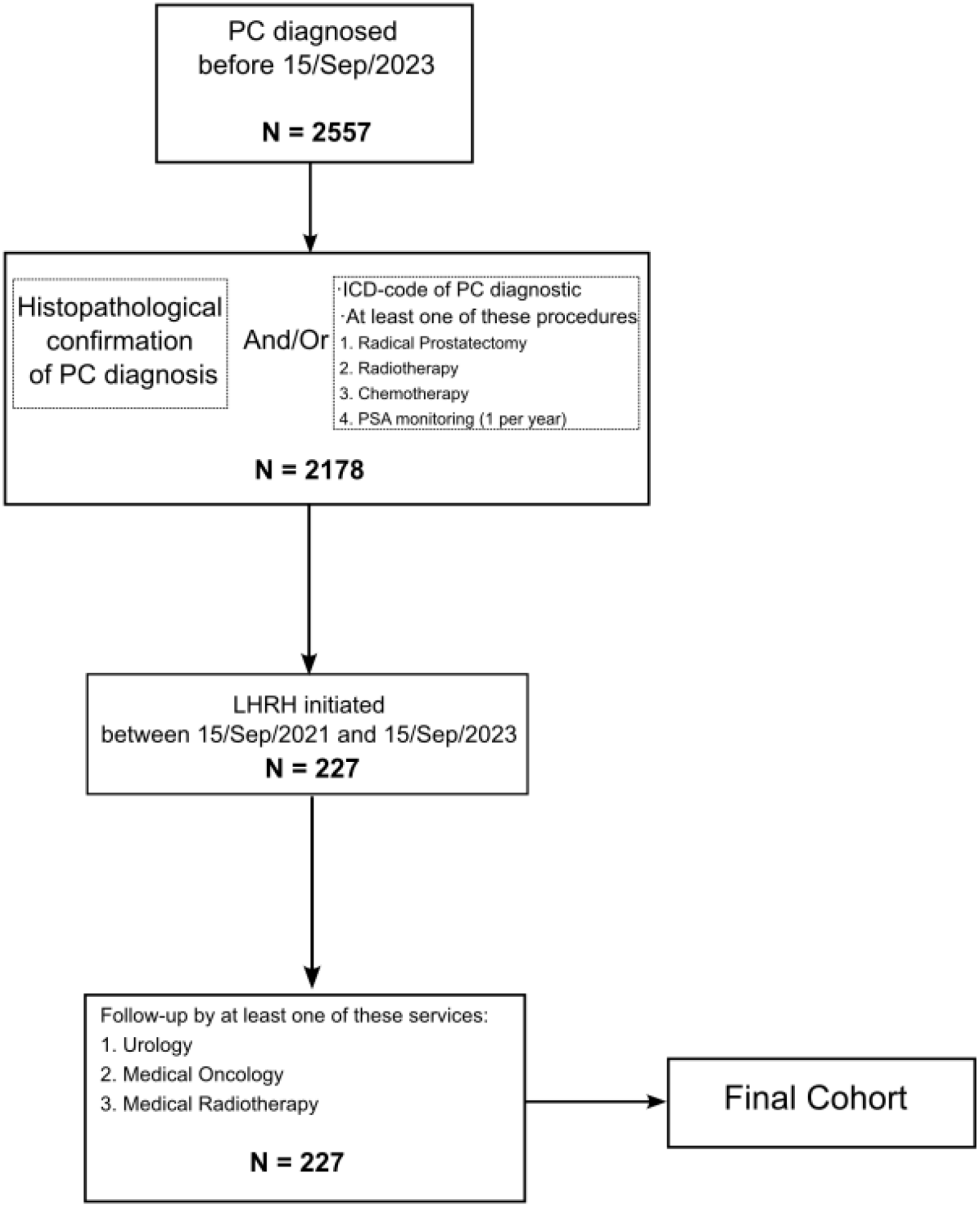
Patient inclusion flowchart. From a total of 2,557 patients diagnosed with prostatic cancer before September 15, 2023, 2,178 met criteria for histopathological confirmation and/or received at least one oncologic treatment or PSA monitoring. Among them, 227 patients-initiated ADT with LHRH analogues between September 15, 2021, and September 15, 2023, and were monitored by Urology, Medical Oncology, or Radiation Oncology services.

A stepwise approach was used to define the analytic cohort. All individuals with a recorded diagnosis of prostate cancer before 15 September 2023 in the institutional data warehouse were screened. Confirmation of diagnosis was required through histopathology or through an ICD code accompanied by evidence of active oncologic management, including radical prostatectomy, external−beam radiotherapy, systemic chemotherapy or sustained PSA surveillance with at least annual testing. From this clinically verified frame, patients who initiated pharmacologic androgen−deprivation with a luteinizing hormone−releasing hormone analogue between 15 September 2021 and 15 September 2023 were retained, while surgical castration was excluded to preserve a uniform treatment modality and a clearly defined index date. Eligibility also required longitudinal care by Urology, Medical Oncology or Radiation Oncology to ensure exposure to the clinical decision support system within routine workflows. The resulting cohort and counts at each step are shown in **Figure 4**.

Across the institutional registry, 5,500 prostate cancer cases were recorded. Of these, 1,831 patients received active oncologic management (TDA), representing 33 percent. Relative to the study window, 1,199 patients initiated TDA before 15 September 2021, 249 initiated TDA after 15 September 2023, and 383 initiated TDA within the interval of interest, representing 21 percent of treated patients. Within the clinically verified frame of 2,178 patients with histopathological confirmation and/or evidence of active oncologic management, 383 patients initiated LHRH−based ADT between 15 September 2021 and 15 September 2023. A total of 156 were excluded because the diagnosis was established outside the hospital without histopathological confirmation in institutional records, or because annual PSA surveillance was not met, or because no surgery, radiotherapy or chemotherapy was recorded to verify active management within the institution. The final analytic cohort comprised 227 patients, equivalent to 60 percent of those initiating TDA within the interval, all with longitudinal follow−up by Urology, Medical Oncology or Radiation Oncology.

### 3.1 Demographic and clinical profile of patients under ADT

A total of 227 PC patients meeting the inclusion criteria were included. **Table 2** provides demographics and clinical information of patients included the study, stratified by Pre-CDSS and Post-CDSS. The table summarizes the number of ADT-treated patients, their age at PC diagnosis and ADT initiation, PSA levels at the time of ADT initiation, and Gleason scores at CP diagnosis. Patients across the two periods were assessed as well balanced at baseline. A slightly younger age at ADT initiation was observed in the post−CDSS period; in the context of similar PSA values at ADT start and a globally comparable distribution of Gleason categories, this difference is not expected to indicate a systematic shift in baseline disease severity. Stability in the mix of initial systemic regimens at the start of ADT further supports comparable treatment intensity at baseline between periods. A complementary multivariable appraisal using baseline PSA, time from diagnosis to ADT and Gleason did not discriminate the periods, which reinforces the interpretation of clinical comparability at treatment start.

**Table 2:** Baseline clinical and demographic characteristics of patients.

Variable	Pre-CDSS	Post-CDSS	p-value; SMD
N patients under ADT (% over PC patients included in the study)	116 (51%)	111 (49%)	
Age at PC diagnosis (Median, IQR)	71.5 (6.17)	70.35 (12.17)	0.08; -0.23
Time from PC diagnosis to ADT initiation (years)	4.72	3.16	0.43; -0.24
Age at ADT initiation (Median, IQR)	76.22 (8.29)	73.51 (13.25)	0.02; -0.31
PSA level at first ADT dose (ng/mL) (Median, IQR)	12.42 (38.75)	18.74 (48.34)	0.40; -0.09
Testosterone level at first ADT dose (ng/dL) (Median, IQR)	53.4 (24.65)	45.9 (14.8)	0.20; 0.08
Gleason score at PC diagnosis biopsy (N, %)			
6	17 (14.65%)	14 (12.61%)	
7	44 (37.93%)	46 (41.44%)	
8	5 (4.3%)	8 (7.2%)	
9–10	10 (8.62%)	17 (15.31%)	
Missing	40 (34.48%)	26 (23.42%)	
Initial systemic regimen at ADT start — counts (n)			Global χ² p=0.87; Fisher p=0.83
ADT alone	(n) — provide	(n) — provide	
ADT + ARPI	24	26	
ADT + RT + ARPI	4	0	
ADT + docetaxel	8	6	
Any intensified regimen (ARPI and/or docetaxel)	Reported as stable across periods	Reported as stable across periods	
Pre−ADT treatments (count)	15	12	
Radical prostatectomy Radiotherapy	67	60	
Chemotherapy	6	6	
Radical prostatectomy and Radiotherapy	13	7	

P-values from Wilcoxon rank-sum tests (continuous variables) and chi-squared or Fisher’s exact tests (categorical variables); SMDs are shown as an additional balance metric. Gleason ‘Missing’ reflects unparsed unstructured pathology; no imputation was performed. ‘Initial systemic regimen’ refers to therapy at ADT start; ‘Any intensified regimen’ denotes ADT plus an ARPI and/or docetaxel.

### 3.2 Impact of CDSS on the PSA instability detection and CPRC progression

**Figure 5:**
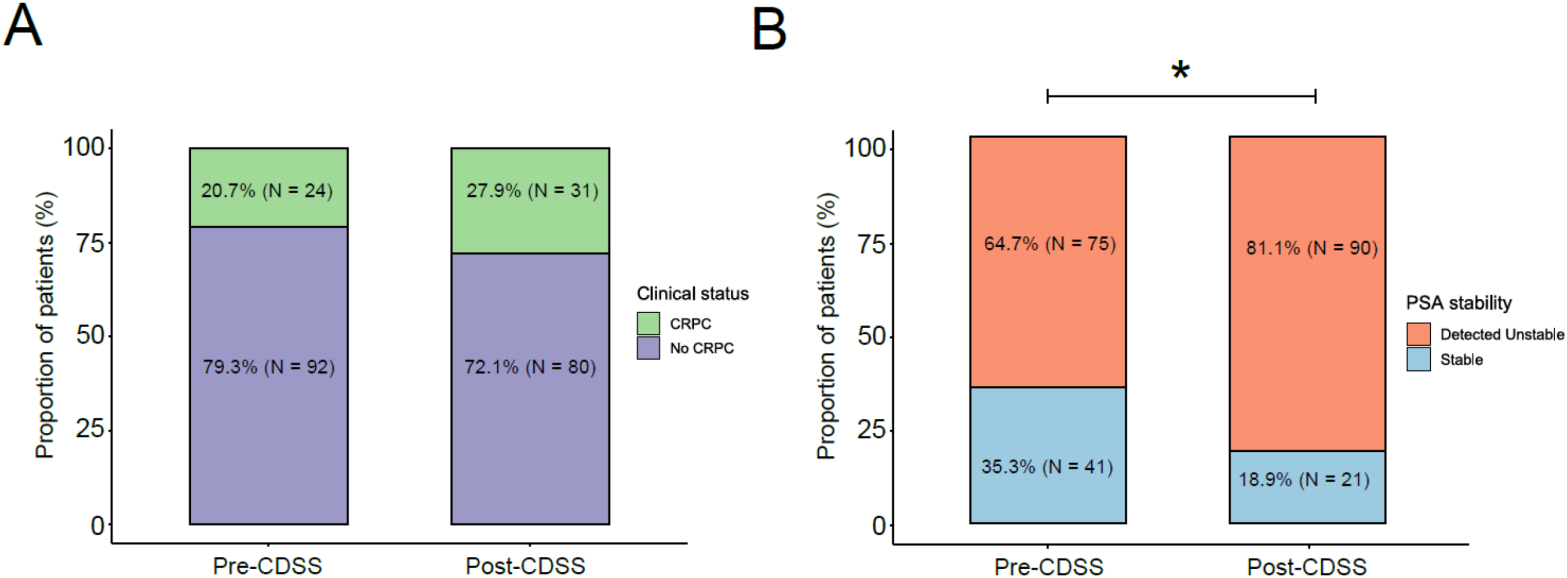
Comparison of CRPC detection and PSA instability before and after CDSS implementation. **(A)** Proportion of patients receiving ADT diagnosed with CRPC in the pre-CDSS (20.7%) and post-CDSS (27.9%) periods. **(B)** Proportion of patients under ADT classified as having unstable PSA kinetics during follow-up (* p<0.01). Percentages and sample sizes (N) are shown within each bar.

Following CDSS deployment, the proportion of patients on ADT classified as CRPC increased, which is consistent with earlier and more consistent application of guideline−aligned rules to signals of progression at the cohort level. The absolute shift remained modest and non−significant within the 12−month post−implementation window, a pattern that is compatible with the study’s sample size and design; we therefore interpret this finding as a process−level improvement in detection and standardization rather than an immediate change in clinical outcomes (**Figure 5A**).

Concordantly, identification of unstable PSA kinetics increased post−CDSS, in keeping with tighter surveillance and more consistent flagging of atypical trajectories, while overall testing intensity remained broadly comparable across periods. This suggests that the gain stems from timeliness and consistency of monitoring rather than from greater test volume (**Figure 5B**). Within this framework, Alert 7, an operational screening flag rather than a progression endpoint, was triggered in 30/227 patients (13.2%), with 137 activations overall; 84/137 (≈61%) occurred while on active ADT (25 patients). This pattern supports the clinical relevance of the signal when interpreted alongside treatment status and testosterone and in complement to the PCWG3−aligned logic specified in **Table 1**, **Figure 2**. Taken together, these results are coherent with the intended function of the dashboard, to standardize follow−up, strengthen risk stratification, and support timely coordination, within the constraints of the observation period.

### 3.3 Impact of CDSS on the use of palliative radiotherapy

**Figure 6:**
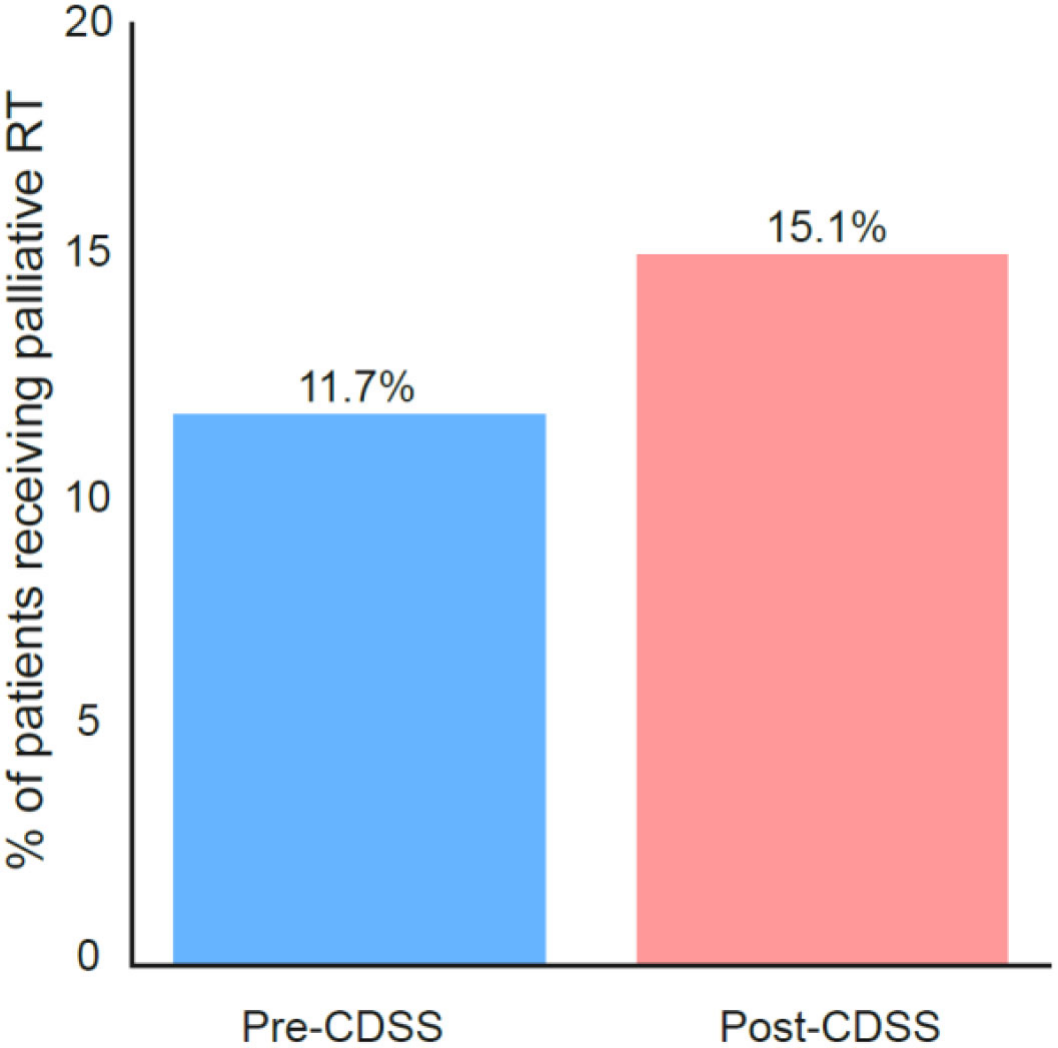
Proportion of patients receiving palliative radiotherapy during the pre- and post-CDSS periods. Pre−CDSS: 9/77 (11.7%); Post−CDSS: 14/93 (15.1%); χ²(1)=0.4, p>0.05.

Use of conventional palliative EBRT was broadly stable across periods, with a slight, non−significant upward tendency after CDSS deployment (**Figure 6**). Given our operational definition (8 Gy × 1, 20 Gy × 5, 30 Gy × 10; all palliative), this endpoint captures symptom−directed courses rather than ablative SBRT. This pattern is compatible with a proximal impact of the CDSS on surveillance and multidisciplinary coordination (e.g., earlier recognition of instability/CRPC) and a more gradual or neutral short−term effect on symptomatic interventions that are primarily triggered by presentation of pain or complications. In addition, the small numbers and the aggregation of radiotherapy indications/targets limit sensitivity to detect changes; longer follow−up and disaggregation by intent/target will better resolve whether the apparent increase translates into a practice signal.

## 4 Discussion

This real-world, retrospective cohort study evaluated the impact of implementing a CDSS on the clinical management of PC patients receiving ADT at La Fe Hospital (Valencia, Spain) between September 15, 2021, to September 15, 2023. The CDSS, developed within the ARCHIMEDES project, was designed to optimize follow-up and clinical decision-making by integrating real-time data visualization, automated alerts based on PSA dynamics and testosterone levels, and adherence to evidence-based guidelines. Its functionality includes generating alerts for unstable PSA patterns, suboptimal castration status, missing tests, and delays in follow-up, thereby facilitating early detection of disease progression and promoting coordinated multidisciplinary action.

The CDSS was designed to address three critical aspects of clinical practice in PC care. It reinforces adherence to evidence-based clinical guidelines by integrating recommendations directly into the clinical workflow, providing automated prompts and recommendations based on individual patient data, thereby reducing variability and promoting standardization across services. In addition, the system enables more precise, risk-adapted monitoring by continuously analyzing clinical parameters such as PSA trends, treatment timelines, and patient characteristics, which allows clinicians to stratify patients by risk and prioritize follow-up accordingly. Furthermore, the tool enhances the management of patients receiving ADT through proactive monitoring and an alert system that notifies clinicians when deviations from recommended schedules occur or when clinically significant changes, such as rising PSA levels, are detected. By addressing these domains, the CDSS supports a more responsive, data-driven approach to patient care, ultimately aiming to reduce delays in critical decision-making and improve outcomes. After its implementation, we observed a trend toward an increased proportion of patients diagnosed with CRPC and a higher proportion classified with unstable PSA kinetics; the between−period difference was statistically significant. There was also a slight, though not statistically significant, increase in the use of palliative radiotherapy. Collectively, these findings suggest that CDSS use was associated with improved monitoring signals and referral to multidisciplinary care. The implications of these results are discussed in detail below.

One important finding was the observed non-significant increase in the proportion of patients on ADT who were diagnosed with CRPC following CDSS implementation. This pattern suggest a more timely and systematic recognition of biochemical progression under routine care. In routine clinical practice, delays in identifying CRPC are common and often stem from inconsistent PSA monitoring, limited integration of patient information, and variability in clinical interpretation (15). Previous observational works has highlighted that inconsistent PSA surveillance and delayed recognition of CRPC can impede timely initiation of evidence-based treatments (16). In this context, the incorporation of automated alerts and real-time monitoring into the CDSS provides structured support for clinicians to recognize rising PSA despite castrate testosterone levels, which define CRPC. This system-driven approach may reduce reliance on subjective clinical judgment and improve adherence to guideline-defined criteria, potentially explaining the increase in detection observed during the post-CDSS period, even within a relatively short observation window.

Another significant finding was the marked increase in the identification of a higher proportion of unstable PSA kinetics after CDSS implementation, while overall testing intensity remained broadly comparable across periods. This shift could reflect the enhanced sensitivity of the CDSS in detecting early signs of disease progression through systematic monitoring and automated alerts. In routine clinical practice, inconsistent PSA testing and variability in interpreting PSA trends often delay recognition of disease progression. A slight increase in the number of PSA tests per patient per year was also observed, from 3.09 to 3.2 tests. Rosinha et al. reported that irregular PSA assessments hinder accurate calculation of PSA doubling time (PSA-DT), a critical parameter for risk stratification in non-metastatic CRPC patients (17). Similarly, Malone et al. found that more than 60% of patients had their PSA measured only twice or fewer in the year preceding CRPC diagnosis, underscoring the challenges of timely detection (18). The integration of automated alerts within the CDSS addresses these limitations by ensuring consistent monitoring and timely identification of instability. Moreover, the system standardizes PSA-DT calculation with a uniform formula embedded within the EHR, promoting consistency across care settings and supporting risk-based decision-making and improves adherence to guideline-recommended care pathways by enabling clinicians to act on objective and reproducible indicators of progression.

Use of conventional palliative EBRT was broadly stable across periods, with a slight, non−significant upward tendency within the current sample and timeframe. Interpretation is limited by the aggregated nature of the RT variable and by our operational endpoint, restricted to canonical palliative schedules (8 Gy × 1, 20 Gy × 5, or 30 Gy × 10) which does not disaggregate intent (palliative vs radical/ablative) or target (primary vs metastasis). Accordingly, we refrain from inferring a practice change and note that larger cohorts, longer follow−up, and explicit separation by intent/target will be required to resolve whether the apparent increase represents a consistent signal.

The direction of effect is consistent with the dashboard’s intended role, strengthening PSA−based surveillance and timely coordination and with contemporary randomized evidence and guidance on appropriate radiotherapy use in advanced prostate cancer (e.g., STAMPEDE, Arm H; PEACE−1). Palliative RT remains central to symptomatic care in advanced disease; our findings are compatible with a setting in which symptom−directed palliation coexists with evolving evidence on disease−modifying roles for prostate irradiation in selected low−volume mHSPC, while overall utilization in this cohort appears broadly stable in the short term.

Several studies have explored the development and implementation of CDSSs for PC, each addressing specific stages or functions within the care continuum. Tools such as the AIPC platform by Siemens Healthineers (19) have demonstrated improvements in decision-making and time efficiency when used by clinicians in controlled environments, primarily in early-stage or screening scenarios. Similarly, CDSS models like the PCT-Map (20) have focused on enhancing the visualization of patient trajectories after radical prostatectomy, offering retrospective interpretation of PSA kinetics and biochemical recurrence patterns. Risk-adapted screening algorithms embedded in EHRs have also shown promise in personalizing PSA testing and improving equity in primary prevention settings (21). However, these tools often operate within narrowly defined contexts, whether diagnostic, post-surgical, or simulated and do not address the complexity of longitudinal follow-up in patients receiving ADT.

In contrast, the CDSS evaluated in this study was embedded in routine clinical workflows and applied across multiple hospital departments, including Urology, Medical Oncology, and Radiation Oncology. By leveraging real-world data from structured EHR sources and implementing rule-based algorithms, the system actively monitored key indicators such as PSA trends, delays in testing, and biochemical progression to CRPC stage. Moreover, it generated real-time alerts that facilitated timely referral between services and improved classification of clinical instability. These capabilities reflect a comprehensive and operationally integrated approach to advanced PC management. To our knowledge, this is one of the first studies to evaluate the clinical impact of a CDSS in the real-world follow-up of ADT-treated PC patients, providing novel insights into its potential to standardize care, reduce delays, and support multidisciplinary coordination.

Several limitations should be considered when interpreting these findings. First, the retrospective and non-randomized design precludes establishing a definitive causal relationship between CDSS implementation and the observed improvements. Although the data reveal a temporal association between the effects and the CDSS deployment, unmeasured confounders such as changes in staffing or variations in patient behavior may have influenced some outcomes. Second, the study was conducted in a single academic hospital with a mature digital infrastructure, which may limit generalizability to institutions with different resources or patient populations. Additionally, the relatively short post-CDSS observation period of 12 months may have restricted the ability to detect long-term effects on long-term clinical outcomes, such as survival or treatment escalation. Another limitation is that the CDSS did not allow clinicians to deactivate or manually manage alerts, which constrained the evaluation of alert fatigue and operational efficiency. Future iterations should incorporate greater flexibility to optimize usability. Finally, the analysis focused on structured clinical and operational data, without assessing patient-centered outcomes such as symptom control, satisfaction, or quality of life, which warrant exploration in future research. Despite these limitations, the present findings offer valuable insights into the potential of CDSS tools to improve risk detection, standardization of follow-up, and timeliness of care in patients with advanced PC. In patients managed with intermittent ADT, off−treatment recovery phases may yield operational positives for Alert 7; clinical review in our workflow explicitly considers testosterone and treatment status when acting on instability signals.

Beyond current biochemical and clinical monitoring, future iterations of the CDSS will evolve alongside precision oncology. Planned developments include integration of theranostic pathways to enable identification of patients potentially eligible for PSMA−targeted radioligand therapy, such as 177Lu−PSMA, as well as incorporation of genomic information, such as BRCA1/2 alterations, to support timely identification of candidates for PARP inhibitors. Such integration would further enhance personalized care and alignment with the evolving standard of care in advanced prostate cancer.

Future studies should explicitly separate palliative, primary−site and metastasis−directed radiotherapy in analytic models and incorporate molecular/imaging variables, such as BRCA status and PSMA−PET referral, that now shape eligibility for targeted therapies and MDT workflows in Europe (14). Mixed-methods approaches incorporating clinician and patient feedback will be essential to assess usability, workflow integration, and real-world effectiveness. Expanding the CDSS to include more granular risk stratification tools, such as genomic data or imaging findings, could further personalize care and enable adaptive follow-up strategies. Ultimately, the continuous refinement of clinical decision support tools, guided by real-world evidence and implementation science, holds the potential to close practice gaps and elevate the standard of care in prostate cancer management.

In conclusion, the implementation of a CDSS within the ARCHIMEDES project was associated with measurable improvements in key aspects of PC care for patients treated with ADT. The system was associated with enhanced identification of patients with PSA instability. These changes suggest that structured, real-time clinical monitoring can support timelier and data-driven decision-making in advanced stages of the disease. Although the increase in the use of palliative radiotherapy was not statistically significant, the overall trends observed are encouraging and align with the system’s intended role in improving adherence to clinical guidelines, harmonizing follow-up strategies, and facilitating multidisciplinary coordination. Beyond these quantitative outcomes, the project also fostered organizational change and heightened awareness of the importance of structured monitoring in advanced disease. Overall, this study demonstrates the feasibility and clinical utility of integrating automated alerts and performance indicators into routine care through a digital platform, even in complex oncological pathways.

## Data Availability

The dataset used in this study was obtained from the institutional Data Warehouse of Hospital La Fe (Valencia, Spain). Due to legal and ethical restrictions related to patient confidentiality and data protection policies, the data cannot be shared publicly. Researchers interested in accessing the data may contact the institution for potential collaboration under appropriate data use agreements. Requests to access the datasets should be directed to Gas ME, eugenia_gas@iislafe.es.
