# Supplementary material for: Monitoring prostate cancer under androgen-deprivation therapy: insights from the implementation of a clinical decision support system dashboard
Source: Front Oncol. 2026 Mar 30;16:1746456. doi: 10.3389/fonc.2026.1746456 (PMC13073089; doi:10.3389/fonc.2026.1746456)
Supplement: Supplementary file 1 [file Table1.docx]

**Supplementary Material**

Supplementary Table 1. ATC Codes for Hormonal Agents Used in the Treatment of Prostate Cancer

| **L02AE – Gonadotropin-Releasing Hormone (GnRH) Analogues** |
| --- |
| L02AE01 – Buserelin |
| L02AE02 – Leuprorelin |
| L02AE03 – Goserelin |
| L02AE04 - Triptorelin |
| L02AE05 – Histrelin |
| **L02BB – Hormone Antagonists and Related Agents** |
| L02BB01 – Flutamide |
| L02BB02 – Nilutamide |
| L02BB03 – Bicalutamide |
| L02BB04 – Enzalutamide |
| L02BB05 – Apalutamide |
| L02BB06 – Darolutamide |
| **L02BX - Other Hormone Antagonists and Related Agents** |
| L02BX02 – Degarelix |
| L02BX03 – Abiraterone |
| **G03HA – Antiandrogens, Single Agents** |
| G03HA01 – Cyproterone |
| **G03HB – Antiandrogens and Estrogens** |
| G03HB01 – Cyproterona y Estrogen |

Supplementary Table 2. ICD-9 and ICD-10 codes for different radiotherapy and brachytherapy modalities included

| **Description** | | **CIE-9** | **CIE-10** |
| --- | --- | --- | --- |
| External Radiotherapy | External Radiotherapy with Photons <1 MeV | 92.24 | DV000ZZ |
|  | External Radiotherapy with Photons 1 to 10 MeV | 92.24 | DV001ZZ |
|  | External Radiotherapy with Photons >10 MeV | 92.24 | DV002ZZ |
|  | Intraoperative Electron External Radiotherapy | 92.41 | DV003Z0 |
|  | External Radiotherapy with Electrons | 92.25 | DV003ZZ |
|  | External Radiotherapy with Heavy Particles (Protons, Ions) | 92.26 | DV004ZZ |
| High-Dose Rate (HDR) Brachytherapy | HDR Brachytherapy with Cesium-137 (Cs-137) | 92.23 | DV1097Z |
|  | HDR Brachytherapy with Iridium-192 (Ir-192) | 92.23 | DV1098Z |
|  | HDR Brachytherapy with Iodine-125 (I-125) | 92.23 | DV1099Z |
|  | HDR Brachytherapy with Other Isotope | 92.23 | DV109YZ |
| Low-Dose Rate (LDR) Brachytherapy | LDR Brachytherapy with Cesium-137 (Cs-137) | 92.23 | DV10B7Z |
|  | LDR Brachytherapy with Iridium-192 (Ir-192) | 92.23 | DV10B8Z |
|  | LDR Brachytherapy with Iodine-125 (I-125) | 92.23 | DV10B9Z |
|  | LDR Brachytherapy with Iodine-125 | 92.23 | DV10BYZ |
| Sterotactic Radiosurgery | Other Type of Stereotactic Radiosurgery with Photons | 92.39 | DV20DZZ |
|  | Stereotactic Radiosurgery with Particles | 92.33 | DV20HZZ |
|  | Stereotactic Radiosurgery with Gamma Rays | 92.32 | DV20JZZ |
| Other Radiation Therapies | Contact Radiation | 92.21 | DVY07ZZ |
|  | Intraoperative Radiotherapy (IORT) | 92.29 | DVY0CZZ |
|  | Single-Source Photon Radiosurgery | 92.31 | DV20JZZ |
| Insertion of Radioactive Element in the Prostate | Insertion into Prostate of Radioactive Element, Open Approach | 92.27 | 0VH001Z |
|  | Insertion into Prostate of Radioactive Element, Percutaneous Approach | 92.27 | 0VH031Z |
|  | Insertion into Prostate of Radioactive Element, Percutaneous Endoscopic Approach | 92.27 | 0VH041Z |
|  | Insertion into Prostate of Radioactive Element, Natural or Artificial Orifice Approach | 92.27 | 0VH071Z |
|  | Insertion into Prostate of Radioactive Element, Natural or Artificial Orifice, Endoscopic Approach | 92.27 | 0VH081Z |

Supplementary Table 3. List of chemotherapy protocols

| **Drug** | **Protocol** |
| --- | --- |
| Docetaxel | Prostate docetaxel 75 mg/m² every 21 days |
| Cabacitaxel | Prostate cabazitaxel 25 mg/m² every 21 days |
| Carboplatin | UC (urothelial cancer) carboplatin AUC 6 every 21 days |
| Mitoxantrone | Prostate mitoxantrone every 21 days |
